# Sexual dimorphism in the walrus mandible: comparative description and geometric morphometrics

**DOI:** 10.7717/peerj.13940

**Published:** 2022-09-20

**Authors:** Mathieu Boisville, Narimane Chatar, Olivier Lambert, Leonard Dewaele

**Affiliations:** 1 Graduate School of Life and Environmental Sciences, University of Tsukuba, Earth Historical Analysis, Earth Evolution Sciences, Tsukuba, Japan; 2 Department of Geology, University of Liège, Evolution & Diversity Dynamics Lab, Liège, Belgium; 3 Royal Belgian Institute of Natural Sciences, Operational Directorate Earth and History of Life, Brussels, Belgium

**Keywords:** Geometric morphometrics, Mandibles, Odobenus rosmarus, Sexual dimorphism

## Abstract

The modern walrus *Odobenus rosmarus* is characterized by marked sexual dimorphism, related to its polygynous behavior and the aggressive competition between males during the breeding season. Previous studies treated skeletal sexual dimorphism in walruses either qualitatively or with basic quantitative measurements. The present study combines a detailed qualitative comparison of male and female walrus mandibles with quantitative two-dimensional geometric morphometrics analysis (principal component analysis, Procrustes ANOVA and a linear discriminant analysis). In addition to identifying previously recognized sexually dimorphic features (e.g., convexity of the anterior margin of the mandible in adult males), our study finds new morphological differences between males and females, such as a relative dorsal expansion of the anterior part of the mandible and an accentuated concavity between the dorsal margin and the coronoid process in adult males. Both our qualitative comparisons and quantitative analyses demonstrate that sexual dimorphism as expressed in the mandible of extant walruses is statistically significant and that (variation in) mandibular morphology can be used as tool to attribute sex with a good degree of accuracy to isolated mandibles or skeletons lacking the cranium. Sexual dimorphism in walruses is directly related to their sexual behavior, characterized as aggressive in males and linked to a polygynous reproduction system. Indeed, the difference in size of the tusks between males and females but also the use of these during intraspecific fights, can reasonably account for this great mandibular morphological disparity between adult males and females, but also among different ontogenetic stages. Finally, the results obtained in the present study may serve as a starting point for assessing sexual dimorphism more in-depth and studying inter- and intraspecific variation in the mandibles of fossil walruses by identifying quantified size and shape mandibular features.

## Introduction

Pinnipeds are a clade of secondarily aquatic carnivoran mammals. Although pinnipeds have streamlined bodies with limbs modified into flippers, allowing for efficient aquatic locomotion, they still retain the ability to move on land, unlike the two other large marine mammal clades (cetaceans and sirenians) (Berta, 2009). Pinnipedia include three extant families: Odobenidae (walruses), Otariidae (sea lions and fur seals), and Phocidae (true seals). With the exception of some otariid and phocid species, most pinnipeds inhabit (sub)polar and temperate regions, particularly the North Atlantic, the North Pacific and the Southern Ocean (*e.g.*, Arnason et al., 2006; Berta, 2009; Berta, Churchill & Boessenecker, 2018; Paterson et al., 2020). Despite their great diversity during the Neogene, Odobenidae are today represented by a single species, the walrus *Odobenus rosmarus*, which has an Arctic circumpolar distribution (Berta, Churchill & Boessenecker, 2018; Lydersen, 2018; Boessenecker & Churchill, 2021).

One of the key morphological characteristics of several pinniped taxa is marked sexual dimorphism, associated with a polygynous behavior (Bartholomew, 1970; Kovacs & Lavigne, 1992; Garlich-Miller & Stewart, 1998; Weckerly, 1998; Lindenfors, Tullberg & Biuw, 2002; Ralls & Mesnick, 2009; Jones & Goswami, 2010; Velez-Juarbe, 2017; Mesnick & Ralls, 2018). For example, in the northern fur seal *Callorhinus ursinus*, adult males may be over five times as heavy as adult females, and in the phocid genus *Mirounga*, adult males may be over three to four times as heavy as adult females (Mesnick & Ralls, 2018). Other expressions of sexual dimorphism in pinnipeds include differences in physical attributes, such as the presence of a proboscis in males of the hooded seal *Cystophora cristata* and the elephant seals *Mirounga* spp., or differences in coat patterns, as in the ribbon seal *Histriophoca fasciata* (Mesnick & Ralls, 2018).

The origin of sexual dimorphism in pinnipeds is debated, although a likely explanation is that sex-related size dimorphism is due to their mating system. Early fossil pinnipeds are proposed to have been characterized by highly ordered polygynous harem systems, guided by the reproductive success of males for females and in association with changing climatic conditions (Cullen et al., 2014).

In *O. rosmarus*, adult males are noticeably larger and heavier than adult females: 3 m and 1,500 kg in average for males, 2.5 m and 900 kg for females (Lydersen, 2018). Over the last few decades, several studies assessed overall size sexual dimorphism in *O. rosmarus* (*e.g.*, Fay, 1982; Kovacs & Lavigne, 1992; Garlich-Miller & Stewart, 1998). Mohr (1942) described sex-dependent morphological differences in the crania and mandibles of *O. rosmarus,* including the spacing between the two tusks, which is proportionally smaller in females; the shape of the anterior margin of the mandible in lateral view, which is straighter in females; and the shape of the mandibular terminus (*i.e.*, corresponding to the anterior tip of the symphysis; Boessenecker & Churchill, 2013) in occlusal view, which is narrower and more pinched in females. Another dimorphic characteristic is the shape of the upper canines or tusks, with males possessing straighter, more divergent, and larger tusks (Fay, 1982). Wiig et al. (2007) and Taylor et al. (2020) presented the ‘Least Mandible Thickness’ (MT), the minimum transverse distance across each hemimandible, posterior to the last postcanine tooth, as a quantifiable, statistically significant difference between male and female walruses. These two studies found that the mandibles of male walruses are proportionally transversely thicker.

However, at least two studies previously assessed sexual dimorphism of the walrus using geometric morphometrics (Jones, Ruff & Goswami, 2013; Randau, Sanfelice & Goswami , 2019), a method which has the potential to further quantify morphological differences between male and female walrus mandibles, moving beyond qualitative descriptions (Bookstein, 1991). Jones, Ruff & Goswami (2013) tested different variables to study the evolution of the shape of the mandible, such as symphyseal morphology, length of the dental row, but also the bite force. Walruses have a unique mandibular morphology among the pinnipeds, especially due to their peculiar tusks and unique behavior and diet.

From a functional point of view, walruses have a different mandible morphology compared to the bearded seal, *Erignathus barbatus*, another suction-feeding pinniped, corresponding to a higher specialization for suction-feeding on bivalve mollusks, and probably related to the robust (pachyostotic) aspect of their mandible (Jones, Ruff & Goswami, 2013). Nonetheless, geometric morphometrics has been used frequently in studies of pinnipeds, to compare cranial morphologies among pinnipeds in relation to their feeding habits (Jones & Goswami, 2010; Jones, Smaers & Goswami, 2015; Kienle & Berta, 2016; Kienle et al., 2018; Randau, Sanfelice & Goswami , 2019; Meloro & Tamagnini, 2021) or their ecological adaptation in relation to ankle morphology in a phylogenetic framework within carnivores (Polly, 2008).

The goals of the present study are to (1) list the major qualitative morphological differences that exist between male and female *O. rosmarus* mandibles; (2) quantitatively describe male and female walrus mandibles through geometric morphometrics in order to identify sources of sexual dimorphism; and (3) test statistically if sex can reliably be identified for an isolated walrus mandible on the basis of its shape. Nevertheless, the present study is also intended to provide a starting point for future work assessing sexual dimorphism also in fossil Odobenidae (such as Kohno & Ray, 2008) by identifying relevant features in the mandible size and shape.

## Material and Methods

### Biological sample

The dataset includes two *Odobenus rosmarus* mandibles from the Institut Royal des Sciences Naturelles de Belgique (IRSNB) and 38 mandibles from the United States National Museum of Natural History (USNM). The sample comprises specimens from various geographical regions, from off Russia to Greenland, through the Bering Strait and Alaska, representing the two currently recognized subspecies (*Odobenus rosmarus divergens* and *O. r. rosmarus*) (Table S1). Therefore, potential differences between subspecies have not been further investigated in this work. IRSNB 1150B and IRSNB 1150D are adult female and male, respectively. Of the 38 USNM specimens, 10 have been identified as males, six as females, and sex was not recorded at collection time for 22 specimens. With the exception of specimens USNM 220151 and USNM 267962, the mandibular symphysis is fused in all specimens. The two specimens from the IRSNB form the basis for the comparative description, with pictures from the USNM specimens used to validate whether or not the morphological differences observed between IRSNB 1150B and IRSNB 1150D are recovered in other males and females of the studied sample.

The ontogenetic stage (adults, subadults, juveniles) has been recorded for each specimen. The proxies used to determine the ontogenetic stage are: the relative size of specimens, tooth wear (especially for canines), the increase in roughness with age of the mandibular condyle, and the reduction of the porosity of the mandibular bone (Fay, 1982; Storå, 2000; Reitz & Wing, 2008). The sample includes 23 adults (including at least ten males and one female), nine subadults (including at least one male and four females) and eight juveniles (including at least two females). Due to the small morphological and size difference between the subadult and adult stages for sexed individuals, subadult individuals were grouped within the adult stage. The complete assignment of each specimen is provided in Table S1.

### Anatomical description

The anatomical terminology used in the present study follows Deméré (1994a); Deméré (1994b) and Kryukova (2012) for the walrus and Evans & deLahunta (2013) for the domestic dog, as a representative for (caniform) Carnivora. Names of mandibular teeth have been abbreviated (incisors = i, canine = c, premolars = p, second premolar = p2, third premolar = p3, fourth premolar = p4, molars = m). TMJ = temporomandibular joint.

### Geometric morphometrics

**Figure 1:**
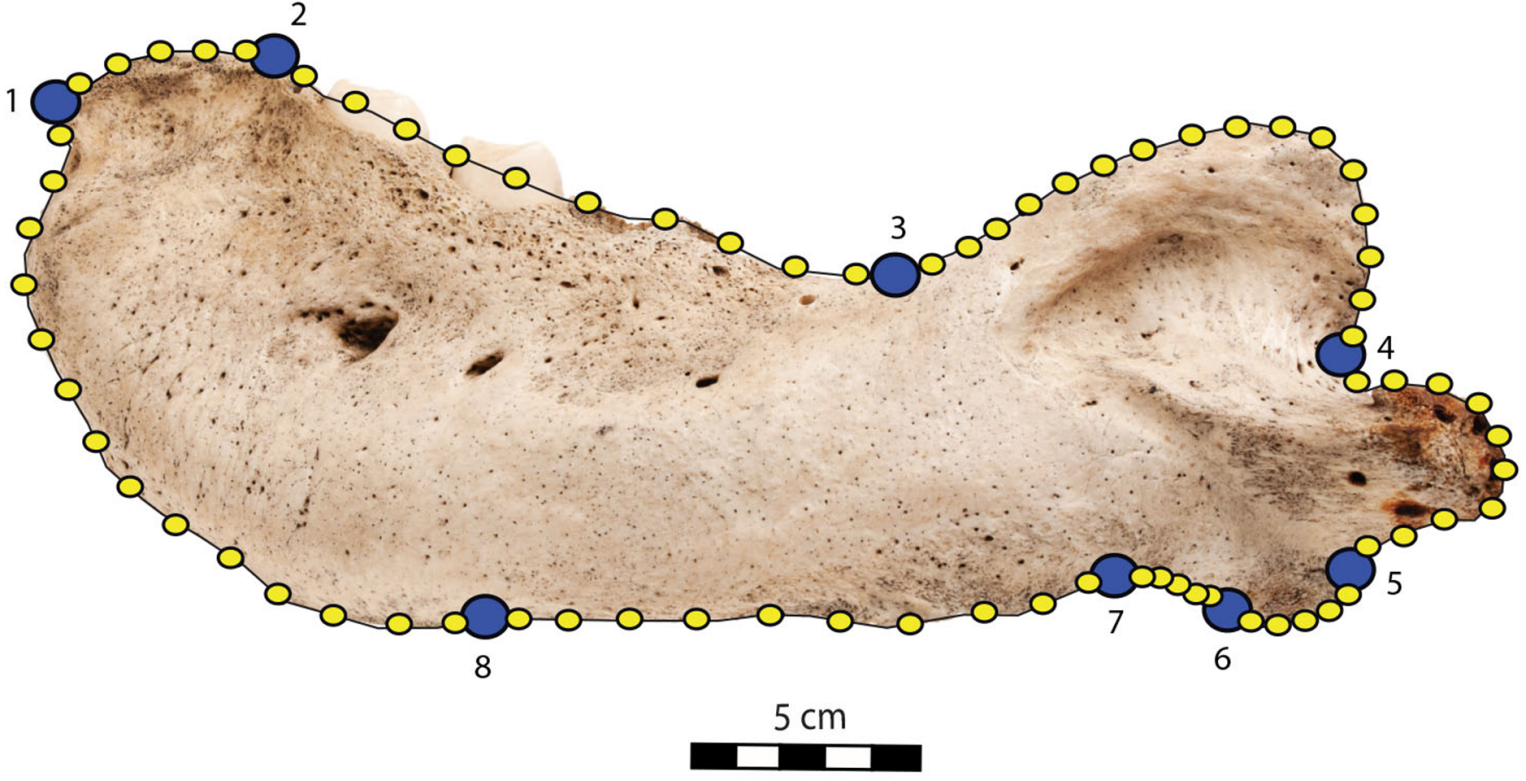
Right mandible of *Odobenus rosmarus* adult male specimen IRSNB 1150D in lateral view (mirrored), showing the position of the eight fixed landmarks (in blue) and the 75 semi-landmarks (in yellow). Scale bar equals 5 cm.

To quantify the shape variation in the mandibles of our sample we used two-dimensional geometric morphometrics, which is the statistical study of morphological variations (shape and size components) through the use of landmark coordinates (Bookstein, 1991; Mitteroecker & Gunz, 2009). We carried on the landmark placement on photographs of the selected specimens in lateral view. Photographs were taken with a Nikon D5300 camera and with a Nikon AF-S DX 18–140 mm f/3.5−5.6G ED VR lens. The camera was located at 50 cm from the specimens, which were positioned with sand and modelling putty to fix them. Eight fixed landmarks were selected and adjoined by 75 semi-landmarks to better capture the mandibular shape (Gunz & Mitteroecker, 2013). Eight curves were placed between the eight fixed landmarks, along which the sliding semi-landmarks were positioned. The number of semi-landmarks per curve was chosen according to the shape and length of the surface to adequately capture the curve shape (Viscosi & Cardini, 2011). The morphological characteristics, represented by the fixed landmarks, are visualized in Fig. 1 and numbered in Table 1.

**Table 1:** List of fixed landmarks applied to the photos of *Odobenus rosmarus* mandibles in lateral view and number of semi-landmarks between sets of two fixed landmarks.

**Landmark**	**Fixed landmarks**	**Number of semi-landmarks between this and the following fixed landmarks**
Mandibular terminus (anteriormost tip of the mandibular terminus)	1–2	5
Dorsal margin (posteriormost tip of the mandibular terminus)	2–3	10
Coronoid process (beginning of the ascending part of the coronoid process)	3–4	16
Mandibular condyle (inflection point between the descending part of the coronoid process and the mandibular condyle)	4–5	10
Angular process (inflection point between the ventralmost part of the mandibular condyle and the posteriormost part of the angular process)	5–6	5
Pre-angular space (between the anteriormost part of the angular process and the posteriormost part of the preangular space)	6–7	5
Ventral margin (between the anteriormost part of the preangular space and the digastric prominence, at the start of the ventral margin)	7–8	10
Anterior margin (between the end of the ventral margin and the genial tuberosity, at the start of the anterior margin)	8–1	14

The landmark coordinates were collected with the tpsDig software (Rohlf, 2006), utility operations were performed using tpsUtil32 (Rohlf, 2018). Landmarks and semi-landmarks raw coordinates are provided in .tps file (Supplemental Dataset S1). Analyses were carried out using the Geomorph and Morpho packages in the R Studio v4.1.0 environment (R Development Core Team, 2008). First, landmark coordinates were imported using the readland.tps function (Rohlf, 2010), then we performed the Generalized Procrust Analysis (GPA) with the gpagen function using bending energy for sliding (Schlager, 2017; Adams, Collyer & Kaliontzopoulou, 2020). To visually assess the morphological differences between males and females we performed a principal component analysis (PCA) using the gm.prcomp function (Baken et al., 2021). After this first visualization two Procrustes ANOVA were carried out on the landmark coordinates with 1,000 permutations using the procD.1m function (Sherratt, 2016). The first one on the sexed specimens only, to determine if sex truly has a significant influence on mandibular shape without considering the age, and then a second on the whole dataset, to test for the influence of the ontogenetic stage (juvenile vs. non-juvenile), not considering the sex. Then, to test for the allometric influence in our dataset we also performed a Procrustes ANOVA to test for the influence of the log centroid size on the shape. Finally, to make sure that sex is actually a good predictor of mandibular shape, we performed a linear discriminant analysis (LDA) using the lda function on the landmark coordinates with a Box’s M-test (Box, 1949) as a prerequisite (Supplemental Dataset S2) on the sexed specimens only. Because of the small size of our dataset we cross validated our LDA results using the ‘CV’ argument of the lda function.

This study includes morphological measurements. These include the measurement of the minimum mandible thickness (MT), following Wiig et al. (2007). The MT is the minimum transverse thickness of the mandible posterior to the last postcanine. The MT has been measured using a digital caliper with an accuracy of ±0.01 mm. Five angular measurements have also been taken, following Mohr (1942) and Deméré (1994a): angle between (a) the anterior and dorsal margin; (b) the anterior and ventral margins; (c) the ventral and dorsal margins; (d) the horizontal and vertical rami; and e) the coronoid process and the mandibular condyle. All measurements are provided as supplemental information (Fig. S1).

## Results

### Comparative description of sexual dimorphism in mandibles of *Odobenus rosmarus*

**Figure 2:**
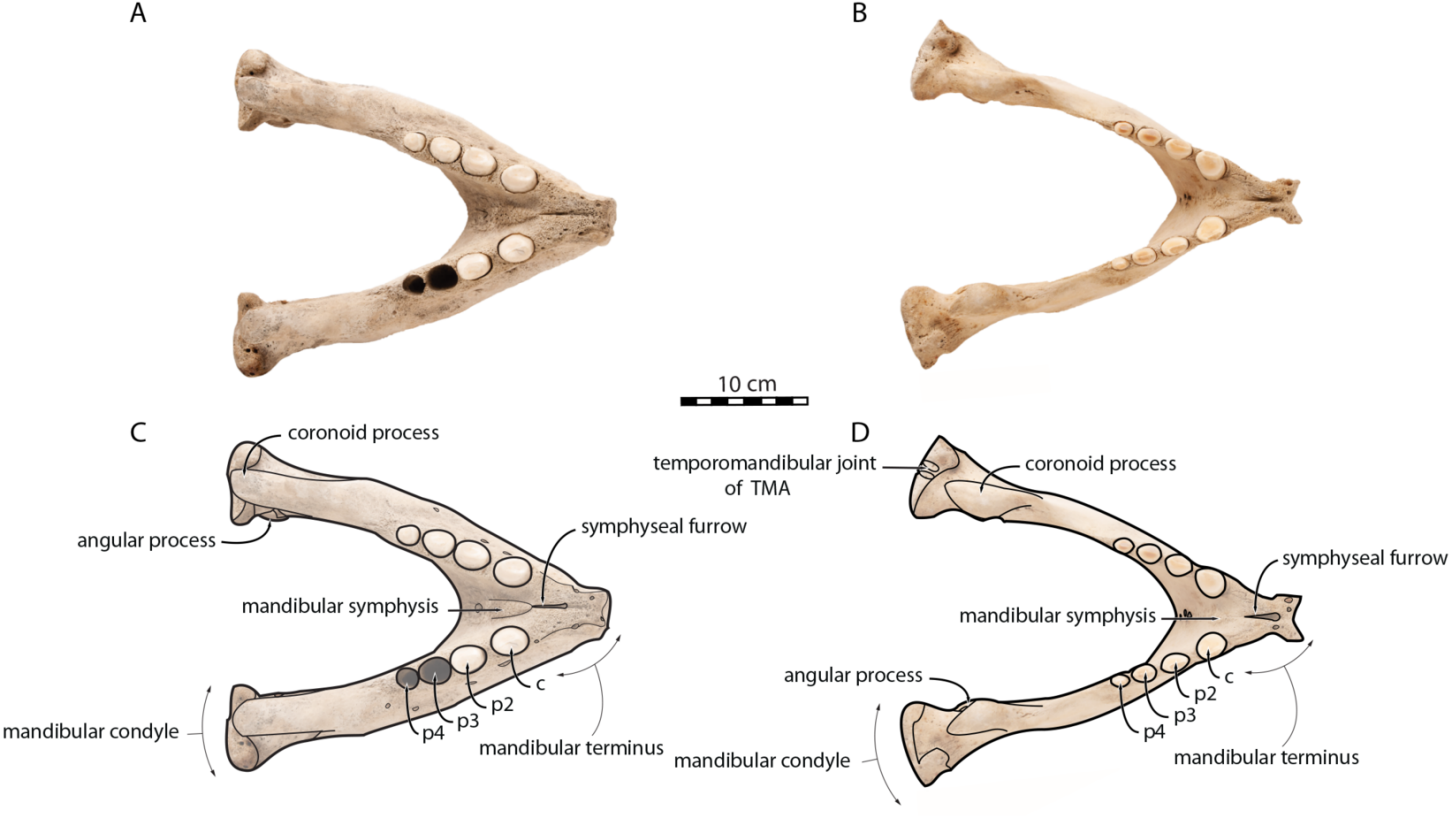
Mandibles of *Odobenus rosmarus* in occlusal view. Adult male specimen IRSNB 1150D (A, C) and adult female specimen IRSNB 1150B (B, D). Scale bar equals 10 cm. TMA = Temporomandibular articulation.

**Figure 3:**
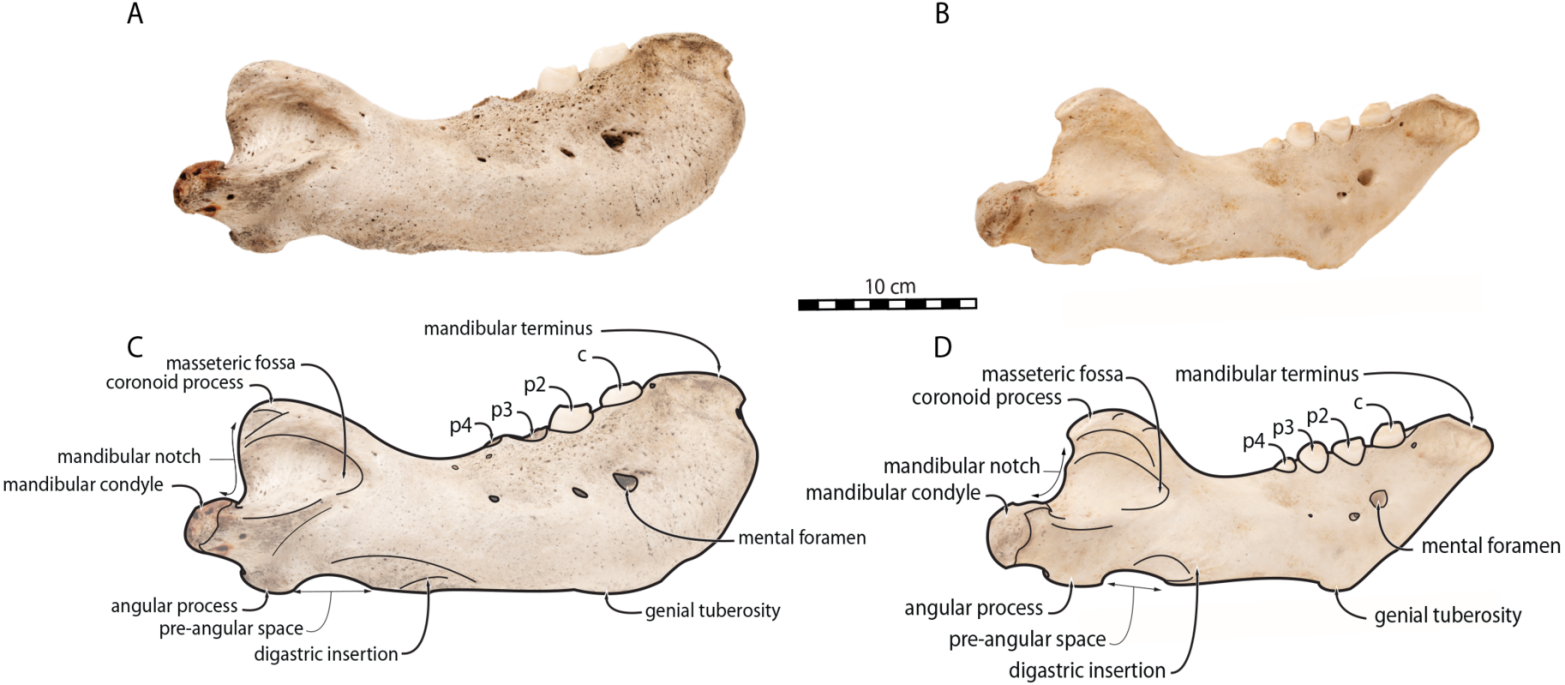
Mandibles of *Odobenus rosmarus* in lateral view. Adult male specimen IRSNB 1150D (A, C) and adult female specimen IRSNB 1150B (B, D). Scale bar equals 10 cm.

*O. rosmarus* has a highly pachyostosic mandible with mandibular dental formula i0c1p3m0. In the absence of lower incisors, the mandibular terminus of *O. rosmarus* is pinched transversely and displays a sagittal symphyseal furrow. The mandibular symphysis is sexually dimorphic: it is transversely thinner in females (Fig. 2). In female walruses, the anterior margin of the mandible is generally straight in lateral view. Towards the mandibular terminus, anterodorsally, this margin becomes abruptly convex (Fig. 3). Adult males differ from females in having a clearly marked convexity of the anterior margin of the mandible in lateral view. Towards the mandibular terminus, this margin is narrowly concave before transitioning to convex in its lower part (Fig. 3). In males, a variable convexity is observed between individuals, with certain—presumably old—adult specimens having a conspicuous rounded anterior margin largely exceeding the anterior tip of the mandibular terminus, as observed by Mohr (1942). The posteroventral margin of the mandibular symphysis corresponds to the genial tuberosity, which is usually the lowest part of the mandible. This tuberosity is better defined in females due to the absence of a swelling for the anterior margin of the mandible (Fig. 3). As a consequence of the important development of the anterior part of the mandible in male walruses, their genial tuberosity expands anterior to the canine and the mandibular terminus. This condition differs from females, in which the genial tuberosity extends anterior to the level of p2. Posterior to the genial tuberosity, the ventral margin of the mandible starts at different levels in males and females: at p2 in males and at p3 in females. Due to the swelling of the anterior margin, males possess a more obtuse angle between the anterior and ventral margins, compared to females. The ventral margin of the mandible is more rectilinear in males (Fig. 3), whereas it is slightly concave in females.

The digastric prominence is located at the posterior end of the ventral margin and serves as the insertion area for the digastric muscle. This prominence is more rugose and robust in males. Due to the strong development and convexity of the anterior part of the mandible in males, the dorsal margin of the horizontal ramus of their mandible is oriented more posterodorsally compared to females. The MT is higher in males compared to females, corroborating previous studies (Wiig et al., 2007; Taylor et al., 2020). The pre-angular space separates the ventral margin anteriorly from the angular process posteriorly. The pre-angular space is located posteriorly to the genial tuberosity and ends at the articular part of the angular process; it is usually ventrally concave. This space is proportionally shorter and more curved posterodorsally, hook-like, in females.

**Figure 4:**
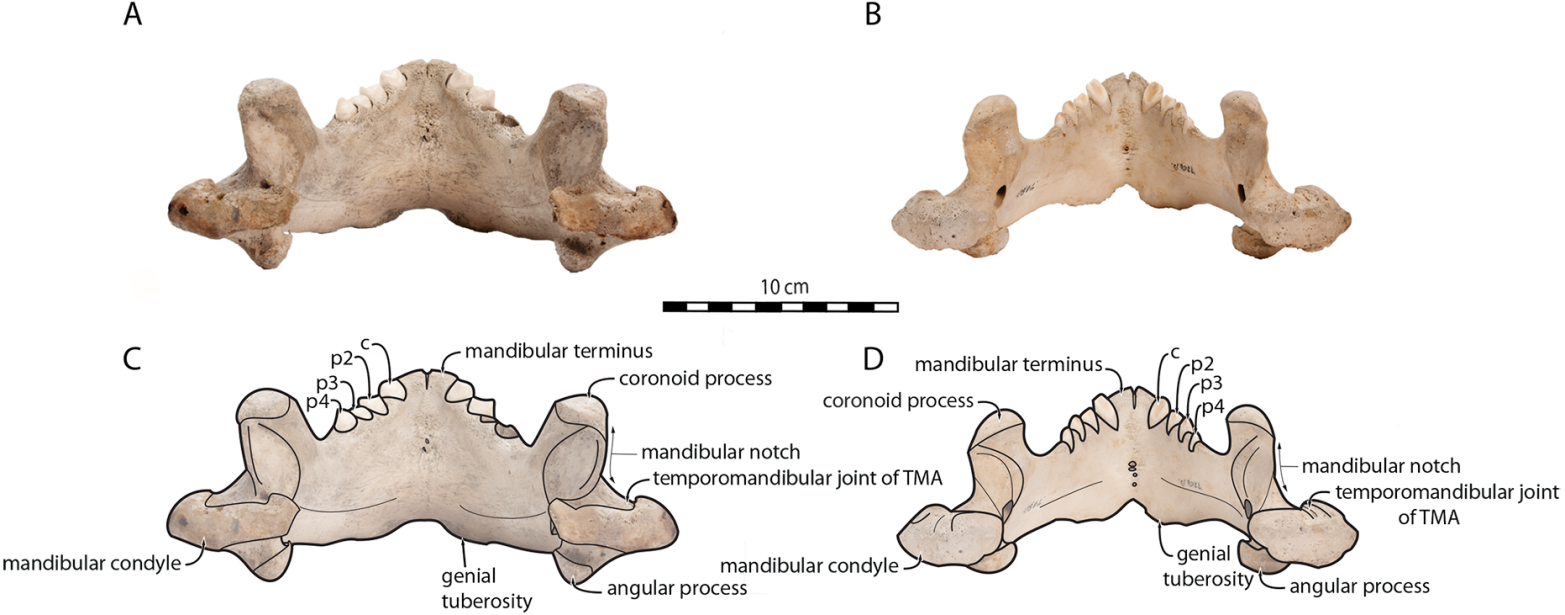
Mandibles of *Odobenus rosmarus* in posterior view. Adult male specimen IRSNB 1150D (A, C) and adult female specimen IRSNB 1150B (B, D). Scale bar equals 10 cm. TMA = Temporomandibular articulation.

**Figure 5:**
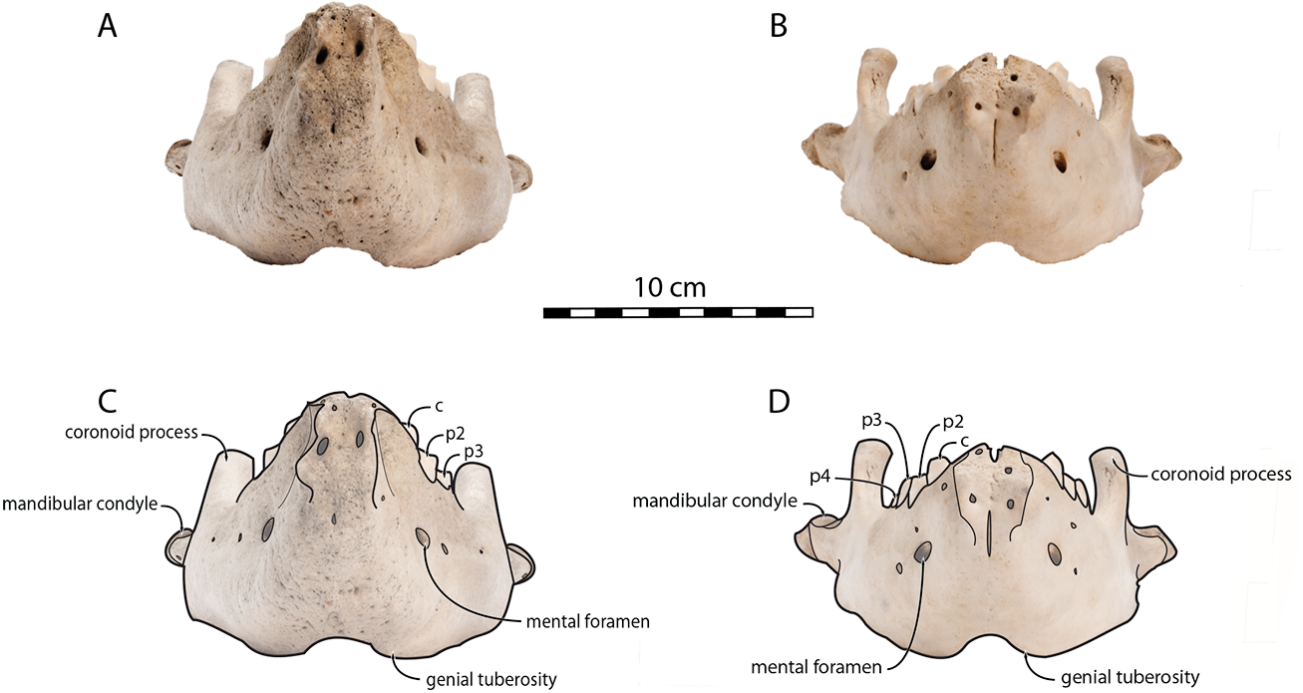
Mandibles of *Odobenus rosmarus* in anterior view. Adult male specimen IRSNB 1150D (A, C) and adult female specimen IRSNB 1150B (B, D). Scale bar equals 10 cm.

Sex-related differences are also noted in the shape of the mandibular condyle. The latter is more elongated posteriorly and more rectangular in posterior view in males; in females, it is more globular and oval. The articular surface of the condyle, connecting the latter with the squamosal, forms a depression where the temporomandibular joint (TMJ) is located (Winer et al., 2016). In males, this depression is located dorsally on the condyle, whereas it is located rather dorsoposteriorly in females (Figs. 3 and 4). Wear of the TMJ is significantly more prevalent in males (Winer et al., 2016). The shape of the mandibular notch, between the mandibular condyle and the coronoid process, varies also between male and female walruses. In males, this notch forms a right or slightly acute angle and the posterior margin of the coronoid process either is vertically straight or slightly overhangs the mandibular notch. In females, the mandibular notch is open, forming an obtuse angle between the mandibular condyle and the coronoid process (Fig. 3), and the posterior margin of the coronoid process is slightly slanted anterodorsally. In females, the coronoid process reaches dorsally a higher level than the mandibular terminus and the anterior part of mandible. On the contrary, due to the important development of the anterior part of the mandible, the coronoid process of males does not reach dorsal to the level of the mandibular terminus, giving a more concave angle between the dorsal margin and the coronoid process (Fig. 3, Table S2). The distance between the last tooth (p4) and the anterior limit of the coronoid process is proportionally greater in females (Fig. 3), where this distance is approximately twice the mesiodistal length of p4, whereas it is closer to the length of p4 in males. On the lateral surface of the coronoid process, the masseteric fossa is deeper in males. A large, oval mental foramen opens antero-laterally on the lateral margin of the horizontal ramus of the mandible of *O. rosmarus*, (Figs. 3 and 5). This foramen is located at the level of c in males and at the level of the diastema between c and p2 in females. Other, smaller mental foramina are present, but their number and position on the mandible varies widely between the specimens of the studied sample, seemingly not following any sexual pattern.

**Figure 6:**
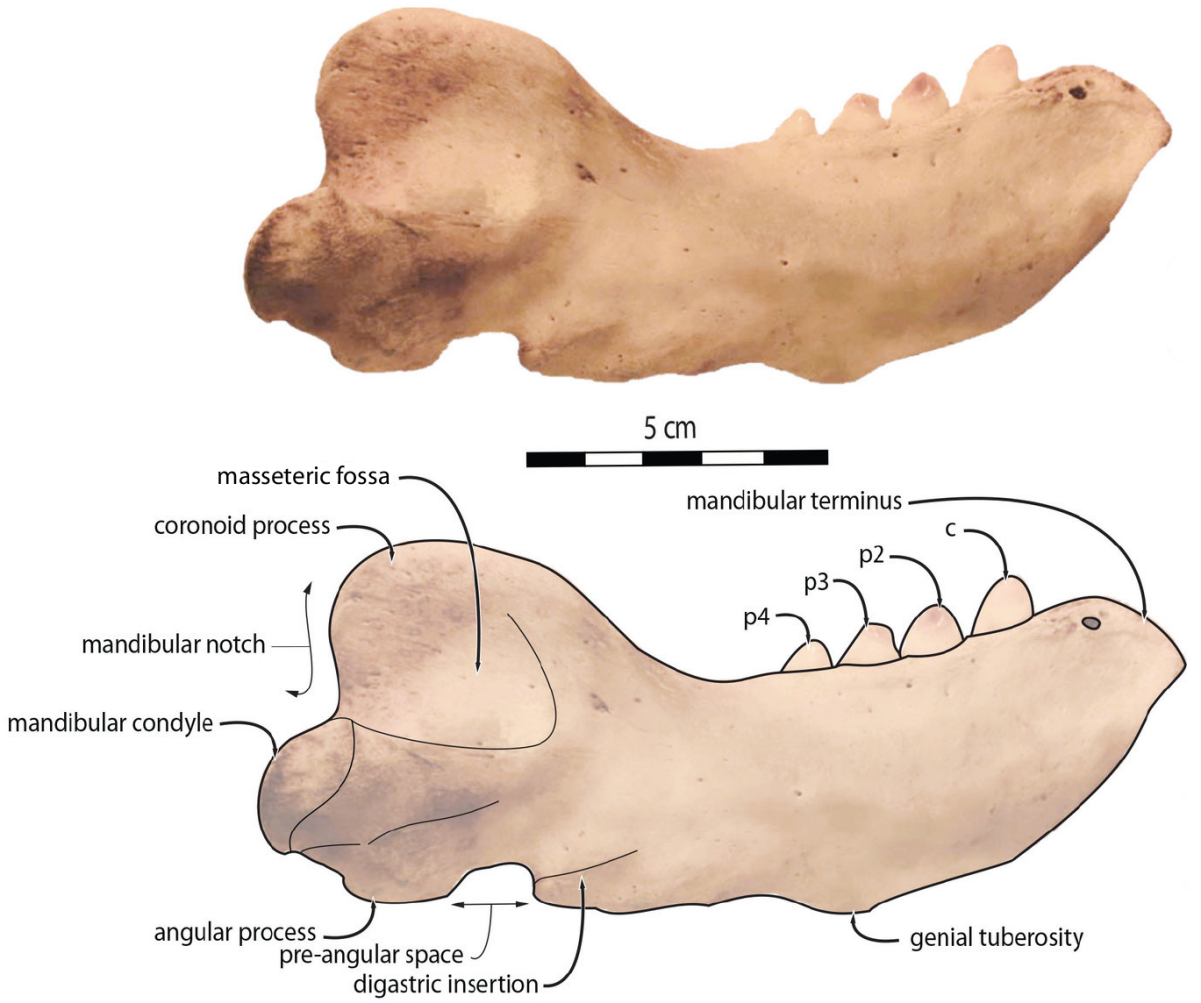
Mandibles of *Odobenus rosmarus* in lateral view. Unsexed juvenile specimen USNM 550408. Scale bar equals 5 cm.

Morphologically, juvenile mandibles from our sample (including 2 females and 6 individuals with no sex identification) have a more sinuous ventral margin and an anterior margin that is not swollen, generally straight in lateral view (one exception is USNM 16445, which displays a slightly concave anterior margin). The genial tuberosity is relatively pronounced, the pre-angular space presents a hook-like form, and the mandibular notch is open, forming an obtuse angle between the mandibular condyle and the coronoid process, with a mandibular condyle being more rounded and the dorsal margin of the horizontal ramus being oriented more anterodorsally (Fig. 6). These characteristics are also found in adult females.

### Geometric morphometrics

First, to visualize the effect of sex on mandibular shape we performed a principal component analysis (PCA) that retrieved 39 components (Fig. S2), the first three axes explaining a total of 64,19% of the morphological variation (32.26%, 19.20%, and 12.73%, respectively).

**Figure 7:**
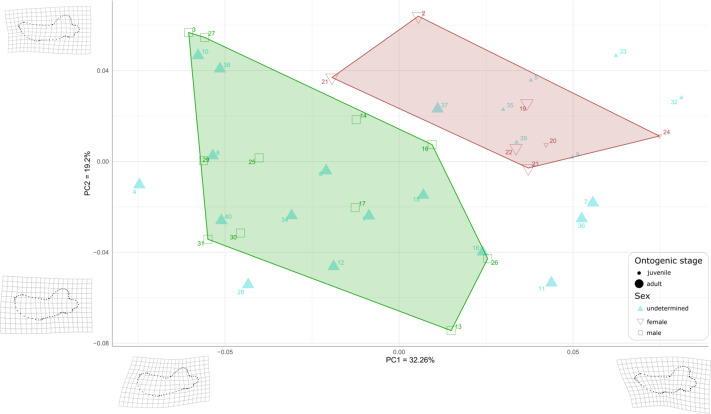
Graph of the PCA for mandibles of *Odobenus rosmarus* for the axes PC1 (on the *x*-axis) and PC2 (on the *y*-axis). **Green square: males of *O. rosmarus*; red inverted triangle: females; blue triangle: sex not determined; small-size dot: juvenile; big-size dot: adult.** Outlines on the lower left represent a male morphotype, with several features such as a straighter ventral margin, a convex anterior margin, and a more concave angle between horizontal and vertical rami. Outlines on the upper right represent a female morphotype, with several features such a slightly concave ventral margin, a straighter anterior margin, and a lower angle between horizontal and vertical rami. (see Table S1 for collection numbers corresponding to each number appearing in the graph).

Males and females are well differentiated on those first two PCs with almost all females showing positive values along both axes (the only exception is USNM 267965 (21), showing negative PC1values and positive PC2 values), resulting in two distinct areas of morphospace occupation for males and females (Fig. 7). Except for USNM 22014 (16), males show negative for either PC1 or PC2, or both. PC1 encompasses the development of the anterior margin, or chin, of the mandible. Higher, positive values of PC1 represent a slightly convex or flat anterior margin of the mandible, and lower, negative, values represent a strongly convex, *i.e.*, bulging, anterior margin. In addition, higher values for PC1 correspond to a more pronounced genial tuberosity, a rather concave pre-angular space, and a rather rounded coronoid process in lateral view, contrasting with a less conspicuous genial tuberosity, a less concave pre-angular space, and slightly more angular coronoid process in lateral view for lower values for PC1. PC2 captures the relative dorsal expansion of the anterior portion of the mandible, with lower values representing proportionally higher anterior portions of the mandible, dorsally exceeding the level of the coronoid process, and higher values representing lower anterior portions of the mandible. In addition, PC2 captures the proportion in size between the horizontal and vertical ramus, with lower values for PC2 corresponding to a shortening of the horizontal ramus and higher values representing an elongation of this part compared to the vertical one.

For the ontogenetic aspect, four juvenile specimens of unknown sex (5, 9, 35, and 39, representing USNM 121177, 16445, 550413, and 7156, respectively) occupy the female area. Two others (32 and 33, representing USNM 550408 and 550409, respectively) are nearby. One juvenile female specimen (24, representing USNM 276625) defines one of the boundaries of the female convex hull, with the highest PC1 value, and the other (20, representing USNM 267963) falls within this hull. No juvenile specimens fall within or close to the convex hull of males.

Ten adult specimens of unknown sex (1, 6, 8, 10, 12, 15, 18, 34, 38 and 40, representing USNM 108344, 14397, 16437, 16446, 16756, 21331, 22200, 550410,7139 and 9475, respectively) fall within the convex hull of males; an eleventh adult specimen, USNM 6780 (37), falls in the area occupied by females.

**Figure 8:**
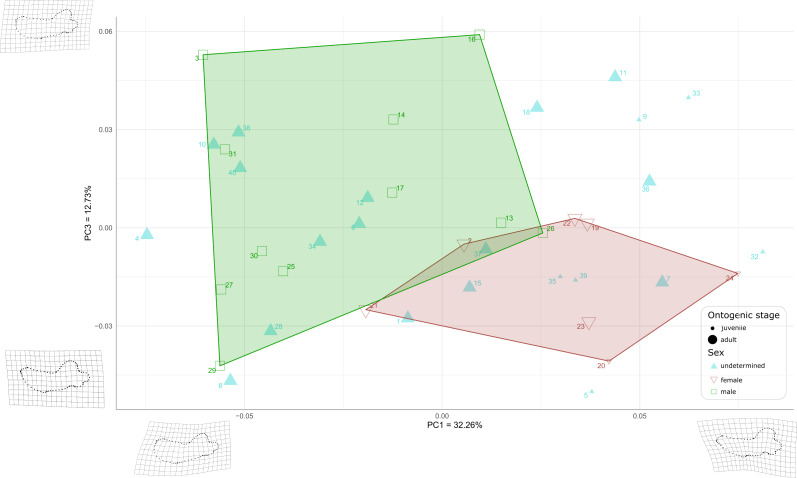
Graph of the PCA for mandibles of *Odobenus rosmarus* for the axes PC1 (on the *x*-axis) and PC3 (on the *y*-axis). **Green square: males of *O. rosmarus*; red inverted triangle: females; blue triangle: sex not determined; small-size dot: juvenile; big-size dot: adult.** Outlines on the upper left represent a male morphotype, with several features such as a straighter ventral margin, a convex anterior margin, and a more concave angle between horizontal and vertical rami. Outlines on the lower right represent a female morphotype, with several features such a slightly concave ventral margin, a straighter anterior margin, and a lower angle between horizontal and vertical rami. (see Table S1 for collection numbers corresponding to each number appearing in the graph).

When plotting PC1 against PC3, the reduction to 44.99% in the percentage of morphological variation explained, due to PC3 explaining only 12.73% of variance, resulted in an overlapping of the areas occupied by males and females (Fig. 8). Females show negative PC3 values except for USNM 267962 (19) and USNM 276030 (22). Males generally occupy lower PC1 values and higher PC3 values than females. The convex hulls of males and females show only little overlap, with IRSNB 1150B (2) being the only female falling within the observed range for males, and USNM 287993 (26) being the only male falling within the range for females. PC3 captures the development and the elongation of the coronoid process posteriorly and the concavity of the angle between the dorsal margin of the mandible and the coronoid process. Lower values for PC3 represent a proportionally less elongated and less posteriorly developed coronoid process. The opposite is true for higher values for PC3. In addition, lower values for PC3 correspond to a more pronounced concavity between the dorsal margin and the coronoid process.

Two juvenile specimens of unknown sex (35 and 39, representing USNM 550413 and 7156, respectively) occupy the female area, and two others (5 and 32, representing USNM 121177 and 550408, respectively) fall just outside the known range of female morphology. Seven adult specimens of unknown sex (6, 10, 12, 28, 34, 38, and 40, representing USNM 14397, 16446, 16756, 35683, 550410, 7139, and 9475 respectively) fall within the male area, and three others (1, 7 and 15, representing USNM 108344, 144995 and 21331, respectively) fall within the female convex hull. One adult specimen, USNM 6780 (37), falls in the area corresponding to the two overlapping hulls. The two juvenile female specimens (20, 24, representing USNM 267963 and USNM 276625, respectively) make two corners of the female field. No juvenile specimens fall within or close to the convex hull of males.

As for the plot showing PC2 against PC3 (36.93% of variation explained, Fig. S2), males and females show more overlap than in the two previous plots, showing thus that males and females are mainly distinguished by PC1.

Following those first graphical results, we performed a Procrustes ANOVA with 1,000 permutations on the sexed specimens to assess if the sex truly has a significant influence on landmark coordinates. We isolated the identified males and females in the dataset to perform this analysis and the *p*-value obtained was far below the significance level (0.001), indicating significant differences between males in females in terms of mandibular shape (Table S3). Then we performed a second Procrustes ANOVA with 1000 permutations to test for statistical differences between adults and juveniles on the whole dataset (males, females and unidentified) and also obtained a significant *p*-value of 0.001, implying an influence of the ontogenetic stage on the mandibular shape. A last Procrustes ANOVA was performed to test for the influence of size (Log centroid size) on the shape and again the *p*-value obtained was significant: 0.001. We performed a principal component analysis (PCA) to obtain graphical representation of this last Procrustes ANOVA with Log centroid size. We observed the same results and arrangements as in our first ANOVA (Fig. S2). As for the influence of allometry in our dataset, our regression between the log centroid size and the first four PCs show different allometric patterns in males and females on PC1 (Fig. S2). Finally, we performed a linear discriminant analysis to test if sex is truly a good predictor of mandibular shape. To do so we randomly split the identified males and females (18 individuals) into a training (80%, 15 individuals) and a test set (20%, three individuals), created a model with the training set and tried to assess the sex of the test set based on this model. The prediction of our model was 100% accurate, our three test specimens were always assigned to the correct sex by our model meaning that sex is an extremely good predictor of mandibular shape. To make sure this was not simply due to the low number of individuals in the test set, we performed other LDAs with increasing ratio of training/test: 70/30 (five test individuals), 60/40 (six test individuals), 50/50 (nine test individuals), and 40/60 (10 test individuals). Our prediction accuracy remained at a 100% until the 50/50 training/test ratio, where it dropped to 87.5%. However, the cross validated error rate was 44%, meaning that these results must be treated carefully. We then computed another linear discriminant analysis without the juvenile specimens and our accuracy remained at 100% until the 30/70 training/test ratio, where it dropped to 90%. Again, the cross validated error rate was high (52%), so those results must be treated carefully.

## Discussion

The comparative description of the male and female walrus mandibles produced a large array of qualitative differences between both sexes; compared to females, males have: (1) a higher concavity of the dorsal margin in lateral view, (2) a straighter ventral margin, (3) a shorter distance between the last tooth and the anterior limit of the coronoid process or (4) a more elongated mandibular condyle. These differences confirm and complete those already identified in previous studies (Mohr, 1942; Wiig et al., 2007; Taylor et al., 2020). Newly observed differences were supported by the geometric morphometrics analysis. These differences were added to the list and include the following adult male features (compared to females): (1) the presence of a significant convexity at the level of the anterior margin of the mandible in lateral view, (2) a genial tuberosity that is less conspicuous, (3) a slightly less angular coronoid process, (4) an important elongation of the horizontal ramus relative to the vertical ramus, and (5) an accentuated concavity between the dorsal margin and the coronoid process. Several other adult male features, such as (6) the non-curvature of the pre-angular space and (7) the relative dorsal expansion of the anterior part of the mandible, were also confirmed. The geometric morphometrics analysis further supports our qualitative observations with a clear distinction of males and females in our PCA based on the first two axes, a significant *p*-value from our Procrustes ANOVA, and extremely high accuracy percentages of our linear discriminant analyses (remaining 100% until 50/50 training/test ratio with all the sample, and remaining 100% until 30/70 training/test ratio without juveniles). Although the geometric morphometrics analysis was not carried out with three-dimensional data, the results provided by the geometric morphometrics analysis confirmed the qualitative observations. Morphological variation captured by PC1 and PC2 confirmed our first-hand observations that in male walruses the anterior margin of the mandible is strongly convex, the genial tuberosity is less conspicuous, and the coronoid process is slightly more angular, as opposed to females.

Furthermore, size difference is also observed between males and females, with an allometric trend more marked in males than in females, although the adjusted R squared are quite low, forcing us to be careful when drawing any conclusions on these regressions.

Thus, this geometric morphometrics analysis also added new morphological characters of adult males, such as the non-curvature of the pre-angular space (covered by PC1) and the relative dorsal expansion of the anterior part of the mandible (covered by PC2), associated with a concavity between the dorsal margin and the coronoid process (covered by PC3). As our Procrustes ANOVA demonstrated that morphological differences between the mandibles of male and female walruses are statistically significant and as LDA showed that sex is a good predictor of mandibular shape, it can be argued that unsexed specimens can be identified as male or female. Based on our observations and the results of our statistical analyses, nine specimens (4, 6, 8, 10, 12, 18, 28, 38, and 40, representing USNM 11746, 14397, 16437, 16446, 16756, 22200, 35683, 7139, and 9475, respectively) could be identified as males, whereas four other specimens (7, 35, 37, and 39, representing USNM 144995, 550413, 6780, and 7156, respectively) could be identified as females. However, cross-validated error rates were high, which suggests that a broader sampling, in terms of total number of specimens, number of specimens by sex and by ontogenetic stage, would be needed to confirm these results.

The results point to the same direction as observations made by Mohr (1942) and Fay (1982), which showed that dimensions of the tusks (upper canines) and the width of the muzzle are correlated. Due to the closer spacing of the tusks in females, the internal distance between the two tusk alveoli is lower in females. As a consequence, this closer spacing limits the space to accommodate the anterior portion of the mandibles in females when closing the mouth. The present study confirms that the anterior portion of the mandible is more gracile and transversely narrower in females. Furthermore, our PCA clearly shows that a robust and strongly convex chin is a distinct male feature in *Odobenus rosmarus*. The continued convexity of the anterior margin of the mandible in males, which is even further marked in older males, fits well with this idea that walrus tusks keep growing throughout their lives (Fay, 1982).

The more important development of the anterior part of the mandible in males may be related to an improved support for larger-sized, heavier tusks in males when the mouth is closed, especially for intraspecific fights as part of a polygynous reproductive system. Indeed, the fact that the mandibular terminus is wider but also shorter in males, make the anterior part of the mandible more robust. In other polygynous pinnipeds, direct fighting can also cause all kinds of lesions and some species, such as the South American sea lion *Otaria byronia* and and the northern elephant seal *Mirounga angustirostris* (Jones, Ruff & Goswami, 2013), have relatively round, large mandibular symphyses that could better resist loads along the dorsoventral and transverse planes, in relation to their aggressive sexual behaviors.

In *Odobenus rosmarus*, antagonistic behaviors are mainly restricted to display, with intimidation attempts between males, especially by vocalizations, control of the position within the harem and visual tusk threat, without physical contacts (Miller, 1975; Sjare & Stirling, 1996). If males do start a fight, the latter is involving contact between tusks, or an intention to touch the face or throat of the opponent with tusks (Miller & Kochnev, 2021). In all cases, the mouth is closed, and the mechanical consequences of these fights are the loads transmitted from the tusks to the mandible, causing damages reflected in differences in the shape of the mandibular condyle but also in changes in the location of the TMJ depression (Winer et al., 2016; Rickert, Kass & Verstraete, 2021). These differences have been confirmed with our sample. Winer et al. (2016) demonstrated elsewhere that lesions compatible with osteoarthritis of the TMJ were frequent. Although furthers research on this pathology is needed, it has been demonstrated that these lesions mostly affect adult males (Fay, 1982; Winer et al., 2016; Rickert, Kass & Verstraete, 2021). In view of our observations and of the recorded sexual behavior in walruses related to a polygynous system, it may be hypothesized that the aggressive use of tusks by males for intraspecific fights during the breeding period may cause lesions on the TMJ, as previously suggested by Winer et al. (2016) and Rickert, Kass & Verstraete (2021). Other, more in-depth, studies on the biomechanical constraints in the walrus mandible and jaw joint are to be carried out in future studies.

Based on the comparative description and geometric morphometrics analyses, the shape of the mandible of juvenile *Odobenus rosmarus* specimens shares many morphological features with adult females, such as a poorly developed and straight anterior margin, a sinuous ventral margin, and a less pronounced genial tuberosity. These results could indicate that the mandibular morphology of females changes little across the different ontogenetic stages. Nevertheless, our sample included a limited number of individuals in the juvenile stage; a higher number of specimens with defined sex from each ontogenetic stage, particularly from the juvenile stage, will allow us to draw more robust conclusions about the contrasted trends during ontogenetic transitions between male and female walruses.

## Conclusions and Perspectives

Following the initial analysis of sexual dimorphism in the mandible of the walrus *Odobenus rosmarus* by Mohr (1942), this study involves a larger sample of 40 mandibles and, for the first time, combines an anatomical comparison with a 2D geometric morphometrics analysis, allowing for a quantification of sex-related differences (and ontogenetic variation) in the mandibular morphology of walruses. The results confirm the great morphological disparity that exists between male and female mandibles in extant walruses. Both our visual observations and geometric morphometrics analysis allowed us to assign the sex by proxy to 13 specimens for which sex was not previously identified. Juvenile mandibles from our sample (including two females and six unsexed individuals) share many characters with the female morphotype.

Differences in the shape of the mandible can be directly related to cranial sexual dimorphism, as morphological differences between males and females at the level of the tusks (thickness, spacing between right and left tusks, and curvature) directly impact the shape of the anterior part of the mandible and as this region is also mechanically strengthened in males. Sexual behaviors (aggressive intraspecific interactions between males and, more generally, a polygynous mating system) could be related to the sex-related differences in modern walrus mandibular morphology, in the same way as in other highly sexually dimorph pinnipeds.

Future studies should also test for differences in mandibular morphology between the Pacific (*Odobenus rosmarus divergens*) and Atlantic subspecies (*Odobenus rosmarus rosmarus)*. In the same manner, this study could serve as the basis of further, larger-scale ontogenetic studies across pinnipeds. Indeed, the tendency towards similar cranial morphologies between juveniles and adult females, is also found in other strongly sexually dimorphic pinnipeds (Jones & Goswami, 2010). This study may also constitute a first step for gaining a better understanding about the appearance and evolution of sexual dimorphism within the Odobenidae lineage, as questioned by Kohno & Ray (2008) and Boessenecker & Churchill (2021). As sexual dimorphism could be related to sexual behavior, and, in the case of the modern walrus, to the polygyny system, the shape of the mandible in extinct walruses could also be studied beyond a feeding ecology point of view.

##  Supplemental Information

10.7717/peerj.13940/supp-1 Supplemental Information 1 List of specimens of *Odobenus rosmarus* used for the geometric morphometrics, with their sex, ontogenetic stage, membership of a subspecies, geographical origin and the number allocated in this study For any additional information concerning USNM specimens, enter the specimen’s collection number in the database: https://collections.nmnh.si.edu/search/mammals/.

10.7717/peerj.13940/supp-2 Supplemental Information 2 TPS file corresponding to numbers and raw coordinates of landmarks and semi-landmarks, assignation number and scale for each specimen

10.7717/peerj.13940/supp-3 Supplemental Information 3 Various scripts used under R Studio for morphometric geometrics analysis to test for significative sexual dimorphism and ontogenetic differences in *Odobenus rosmarus* (1) Script used under R Studio for morphometric geometrics analysis to test for significative sexual dimorphism and ontogeny in *Odobenus rosmarus* by Procrustes ANOVA.
(2) Script used under R Studio for morphometric geometrics analysis to test for significative sexual dimorphism and ontogenetic differences in *Odobenus rosmarus* by linear discriminant analysis.
(3) Script used under R studio for morphometric geometrics analysis to observe allometric trends in *Odobenus rosmarus* with Log Centroid Size.

10.7717/peerj.13940/supp-4 Supplemental Information 4 Angle measurements on mandibles of adult female *Odobenus rosmarus* 1150B in lateral view (A, C) and occlusal view (B) Angle between a) the anterior and dorsal margin; b) the anterior and ventral margins; c) the ventral and dorsal margins; d) the horizontal and vertical ramis; and e) the coronoid process and the mandibular condyle. MT: Least Mandible Thickness.

10.7717/peerj.13940/supp-5 Supplemental Information 5 Notable measurements of *Odobenus rosmarus* specimens IRSNB 1150B (♂) and IRSNB 1150D (♂), in mm Letters of angles and measures correspond to those indicated in Fig. S1

10.7717/peerj.13940/supp-6 Supplemental Information 6 Various graphs correspond to the percentage variation (1), shape of the average mandible (2), PC2-PC3 (3), PC1-PC2 with Log Centroid Size (4) and regression between log centroid size and PCs (5) (1) Barplot showing the percentage of morphological variation explained by each of the 39 axes of the PCA.
(2) Graphic representation of the general shape of the average mandible with Generalized Procrust Analysis.
(3) Graph of the PCA for Odobenus rosmarus for the axes PC2 (on the x-axis) and PC3 (on the y-axis). Green square: males of O. rosmarus; red inverted triangle: females; blue triangle: sex not determined; small-size dot: juvenile; big-size dot: adult. Outlines on the upper left represent a morphotype, with features such as a shorter horizontal ramis and a significant more concave angle between horizontal vertical ramis, different from the male morphotype expressed for the lowest value of PC1 & PC2 (Figs. 7 and 8). Outlines on the lower right represent a another morphotype, with features such as a extended horizontal ramis compared to the vertical ones and a straighter ventral margin, different from the female morphotype for the highest value of PC1 & PC2 (Figs. 7 and 8). The numbers assigned to specimens follow the order etablished for the *O. rosmarus* specimens (Table S1).
(4) Graph of the PCA for Odobenus rosmarus for the PC1 (on the x-axis) and PC2 (on the y-axis) using Log Centroid Size. Green square: males of *O. rosmarus*; red inverted triangle: females; blue triangle: sex not determined; small-size dot: 60; medium-size dot: 80; big-size dot: 100. Outlines on the lower left represent a male morphotype, with several features such as a straighter ventral margin, a convex anterior margin, and a more concave angle between horizontal and vertical rami. Outlines on the upper right represent a female morphotype, with several features such a slightly concave ventral margin, a straighter anterior margin, and a lower angle between horizontal and vertical rami. The numbers assigned to specimens follow the order etablished for the *O. rosmarus* specimens (Table S1).
(5) Graph representation of regression between the log centroid size and the first fourth components of PCAs. Green square: males of *O. rosmarus*; red inverted triangle: females; small-size dot: 60; medium-size dot: 80; big-size dot: 100. Centroid size of the specimen was defined as variable for the point size.

10.7717/peerj.13940/supp-7 Supplemental Information 7 Summary table of results of Procrustes ANOVA Hypothesis H0: There is no impact of sex on the shape of the mandible

## Institutional abbreviations

IRNSB: Institut Royal des Sciences Naturelles de Belgique, Brussels, Belgium
USNM: National Museum of Natural History, Smithsonian Institution, Washington, D.C., U.S.A.

## Additional Information and Declarations

### Data Availability

The following information was supplied regarding data availability: The raw data is available in the Supplementary Files.

## References

[ref-1] Adams D, Collyer M, Kaliontzopoulou A (2020). https://cran.r-project.org/package=geomorph.

[ref-2] Arnason U, Gullberg A, Janke A, Kullberg M, Lehman N, Petrov EA, Väinölä R (2006). Pinniped phylogeny and a new hypothesis for their origin and dispersal. Molecular Phylogenetics and Evolution.

[ref-3] Baken EK, Collyer ML, Kaliontzopoulou A, Adams DC (2021). geomorph v4.0 and gmShiny: enhanced analytics and a new graphical interface for a comprehensive morphometric experience. Methods in Ecology and Evolution.

[ref-4] Bartholomew GA (1970). A model for the evolution of pinniped polygyny. Evolution.

[ref-5] Berta A, Wursig B, Perrin W, Thewissen JGM (2009). Pinniped evolution. Encyclopedia of marine mammals.

[ref-6] Berta A, Churchill M, Boessenecker RW (2018). The origin and evolutionary biology of pinnipeds: seals, sea lions, walruses. Annual Review of Earth and Planetary Sciences.

[ref-7] Boessenecker RW, Churchill M (2013). A reevaluation of the morphology, paleoecology, phylogenetic relationships of the enigmatic walrus pelagiarctos. PLOS ONE.

[ref-8] Boessenecker RW, Churchill M, Keighley X, Olsen MT, Jordan P, Desjardins SPA (2021). Chapter 2: The surprising evolutionary heritage of the Atlantic walrus as chronicled by the fossil record. Atlantic Walrus: multidisciplinary insights into human–animal interactions.

[ref-9] Bookstein FL (1991). Morphometric tools for landmark data.

[ref-10] Box GEP (1949). A general distribution theory for a class of likelihood criteria. Biometrika.

[ref-11] Cullen TM, Fraser D, Rybczynski N, Schroder-Adams C (2014). Early evolution of sexual dimorphism and polygyny in Pinnipedia. Evolution.

[ref-12] Deméré TA (1994a). Two new species of fossil walruses (Pinnipedia: Odobenidae) from the Upper Pliocene San Diego Formation. California. Proceedings of the San Diego Society of Natural History.

[ref-13] Deméré TA (1994b). The family Odobenidae: a phylogenetic analysis of fossil and living taxa. Proceedings of the San Diego Society of Natural History.

[ref-14] Evans HE, deLahunta A (2013). Miller’s anatomy of the dog.

[ref-15] Fay FH (1982). Ecology and Biology of the Pacific Walrus, *Odobenus rosmarus divergens* Illiger. United States Department of the Interior, Fish and Wildlife Service, North American Fauna.

[ref-16] Garlich-Miller JL, Stewart REA (1998). Growth and sexual dimorphism of Atlantic walrus (*Odobenus rosmarus rosmarus*) in Foxe Basin, Northwest Territories, Canada. Marine Mammal Science.

[ref-17] Gunz P, Mitteroecker P (2013). Semilandmarks: a method for quantifying curves and surfaces. Hystrix.

[ref-18] Jones KE, Goswami A, Goswani A, Friscia A (2010). Chapter 12: morphometric analysis of cranial morphology in pinnipeds (Mammalia, Carnivora): convergence, ecology, ontogeny, dimorphism. Carnivoran evolution: new views on phylogeny, form and function.

[ref-19] Jones KE, Ruff CB, Goswami A (2013). Morphology and biomechanics of the pinniped jaw: mandibular evolution without mastication. The Anatomical Record.

[ref-20] Jones KE, Smaers JB, Goswami A (2015). Impact of the terrestrial-aquatic transition on disparity and rates of evolution in the carnivoran skull. BioMed Central Evolutionary Biology.

[ref-21] Kienle SS, Berta A (2016). The better to eat you with: the comparative feeding morphology of phocid seals (Pinnipedia, Phocidae). Journal of Anatomy.

[ref-22] Kienle SS, Hermann-Sorensen H, Costa DP, Reichmuth C, Mehta RS (2018). Comparative feeding strategies and kinematics in phocid seals: suction without specialized skull morphology. The Journal of Experimental Biology.

[ref-23] Kohno N, Ray CE (2008). Pliocene walruses from the Yorktown Formation of Virginia and North Carolina, a systematic revision of the North Atlantic Pliocene walruses. Virginia Museum of Natural History Special Publication.

[ref-24] Kovacs KM, Lavigne DM (1992). Maternal investment in Otariid seals and walruses. Canadian Journal of Zoology.

[ref-25] Kryukova NV (2012). Dentition in Pacific walrus (*Odobenus rosmarus divergens*) calves of the year. Biology Bulletin.

[ref-26] Lindenfors P, Tullberg B, Biuw M (2002). Phylogenetic analyses of sexual selection and sexual size dimorphism in pinnipeds. Behavioral Ecology and Sociobiology.

[ref-27] Lydersen C, Wursig B, Thewissen JGM, Kovacs K (2018). Walrus. Encyclopedia of marine mammals.

[ref-28] Meloro C, Tamagnini D (2021). Macroevolutionary ecomorphology of the Carnivora skull: adaptations and constraints in the extant species. Zoological Journal of the Linnean Society.

[ref-29] Mesnick S, Ralls K (2018). Sexual dimorphism. Encyclopedia of marine mammals.

[ref-30] Miller EH (1975). Walrus ethology. I. The social role of tusks and applications of multidimensional scaling. Canadian Journal of Zoology.

[ref-31] Miller EH, Kochnev AA, Campagna C, Harcourt R (2021). Chapter 22: ethology and behavioral ecology of the Walrus (*Odobenus rosmarus*) with emphasis on communication and social behavior. Ethology and behavioral ecology of otariids and the odobenid of ethology and behavioral ecology of marine mammals series.

[ref-32] Mitteroecker P, Gunz P (2009). Advances in geometric morphometrics. Evolutionary Biology.

[ref-33] Mohr E (1942). Geschlechtsunterschiede am WalroszSchàdel. Zoologischer Anzeiger.

[ref-34] Paterson RS, Rybczynski N, Kohno N, Maddin HC (2020). A total evidence phylogenetic analysis of pinniped phylogeny and the possibility of parallel evolution within a monophyletic framework. Frontiers in Ecology and Evolution.

[ref-35] Polly PD, Sargis E, Dagosto M (2008). Adaptive zones and the pinniped ankle: a 3D quantitative analysis of carnivoran tarsal evolution. Mammalian evolutionary morphology: a tribute to Frederick S. Szalay.

[ref-36] Ralls K, Mesnick S, Wursig B, Perrin W, Thewissen JGM (2009). Sexual dimorphism. Encyclopedia of marine mammals.

[ref-37] Randau M, Sanfelice A, Goswami D (2019). Shifts in cranial integration associated with ecological specialization in pinnipeds (Mammalia, Carnivora). Royal Society Open Science.

[ref-38] R Development Core Team (2008). https://www.r-project.org/.

[ref-39] Reitz EJ, Wing ES, Reitz EJ, Wing ES (2008). Size and age in animals with determinate growth. Zooarcheology. Cambridge manuals in archaeology.

[ref-40] Rickert SS, Kass PH, Verstraete FJM (2021). Temporomandibular joint pathology of wild carnivores in the Western USA. Frontiers in Veterinary Science.

[ref-41] Rohlf FJ (2006). tpsDig, digitize landmarks and outlines.

[ref-42] Rohlf FJ (2010). tpsRelw: relative warps analysis.

[ref-43] Rohlf FJ (2018). tpsUtil32 version 1.70. http://life.bio.sunysb.edu/morph/.

[ref-44] Schlager S, Zheng G, Li S, Székely G (2017). Morpho and Rvcg –Shape Analysis in R: R-package for geometric morphometrics, shape analyses and surface manipulation. Statistical shape and deformation analysis: methods, implementation and applications.

[ref-45] Sherratt E (2016). http://www.emmasherratt.com/uploads/2/1/6/0/21606686/quick_guide_to_geomorph-_introduction.html.

[ref-46] Sjare B, Stirling I (1996). The breeding behavior of Atlantic walruses, Odobenus rosmarus rosmarus in the Canadian High Arctic. Canadian Journal of Zoology.

[Storå(2000] Storå J (2000). Skeletal development in the Grey seal *Halichoerus grypus*, the Ringed seal *Phoca hispida botnica*, the Harbour seal *Phoca vitulina vitulina*, the Harp seal *Phoca groenlandica*, Epiphyseal fusion and life history. Archaeozoologia.

[ref-48] Taylor N, Clark CT, Misarti N, Horstmann L, Hohn A (2020). Determining sex of adult Pacific walruses from mandible measurements. Journal of Mammalogy.

[ref-49] Velez-Juarbe J (2017). Eotaria citrica, sp. nov. a new stem otariid from the Topanga formation of Southern California. PeerJ.

[ref-50] Viscosi V, Cardini A (2011). Leaf morphology, taxonomy and geometric morphometrics: a simplified protocol for beginners. PLOS ONE.

[ref-51] Weckerly FW (1998). Sexual-size dimorphism: influence of mass and mating systems in the most dimorphic mammals. Journal of Mammalogy.

[ref-52] Wiig Ø, Born I, Gjertz C, Lydersen REA, Stewart EW (2007). Historical sex-specific distribution of Atlantic walrus (*Odobenus rosmarus rosmarus*) in Svalbard assessed by mandible measurements. Polar Biology.

[ref-53] Winer JN, Arzi B, Leale DM, Kass PH, Verstraete FJM (2016). Dental and temporomandibular joint pathology of the walrus (*Odobenus rosmarus*). Journal of Comparative Pathology.

