# Supplementary material for: Sexual dimorphism in the walrus mandible: comparative description and geometric morphometrics
Source: PeerJ. 2022 Sep 20;10:e13940. doi: 10.7717/peerj.13940 (PMC9504446; doi:10.7717/peerj.13940)
Supplement: Supplemental Information 1 — For any additional information concerning USNM specimens, enter the specimen’s collection number in the database: https://collections.nmnh.si.edu/search/mammals/. [file peerj-10-13940-s001.docx]

| specimen | sex | ontogenetic stage | subspecies | geographical origin | number |
| --- | --- | --- | --- | --- | --- |
| USNM 108344 | NA | A | *O. r. laptevi* | Siberia East Cape, Russia | 1 |
| IRSNB 1150B | F | A | NA | NA | 2 |
| IRSNB 1150D | M | A | NA | NA | 3 |
| USNM 11746 | NA | A | *O. r. divergens* | St Paul Island, Alaska, US | 4 |
| USNM 121177 | NA | J | *O.r. laptevi* | Franz Josef Land, Russia | 5 |
| USNM 14397 | NA | A | *O. r. divergens* | Point Barrow, Alaska, US | 6 |
| USNM 144995 | NA | SA | *O. r. divergens* | NA | 7 |
| USNM 16437 | NA | A | *O. r. laptevi* | Providence Bay, Est Siberia, Russia | 8 |
| USNM 16445 | NA | J | *O. r. laptevi* | Big Diomede Island, Artic Region, Russia | 9 |
| USNM 16446 | NA | A | *O. r. laptevi* | Big Diomede Island, Artic Region, Russia | 10 |
| USNM 16447 | NA | SA | *O. r. laptevi* | Big Diomede Island, Artic Region, Russia | 11 |
| USNM 16756 | NA | A | *O. r. divergens* | Point Barrow, Alaska, US | 12 |
| USNM 200336 | M | A | *O. r. divergens* | Walrus Island, Alaska, US | 13 |
| USNM 21044 | M | A | *O. r. divergens* | NA | 14 |
| USNM 21331 | NA | A | *O. r. divergens* | Bering Island, Alaska, US | 15 |
| USNM 22014 | M | A | NA | Ungava Bay, Canada | 16 |
| USNM 220151 | M | A | *O. r. divergens* | Round Island, Alaska, US | 17 |
| USNM 22200 | NA | A | *O. r. divergens* | NA | 18 |
| USNM 267962 | F | SA | NA | Smith Sound, NW Greenland | 19 |
| USNM 267963 | F | J | *O. r. rosmarus* | Smith Sound, NW Greenland, | 20 |
| USNM 267965 | F | SA | NA | Smith Sound, NW Greenland | 21 |
| USNM 276030 | F | SA | *O. r. divergens* | St Paul Island, Alaska, US | 22 |
| USNM 276624 | F | SA | *O. r. rosmarus* | Greenland | 23 |
| USNM 276625 | F | J | NA | Greenland | 24 |
| USNM 287992 | M | A | *O. r. divergens* | Round Island, Alaska, US | 25 |
| USNM 287993 | M | A | *O. r. divergens* | Round Island, Alaska, US | 26 |
| USNM 291842 | M | A | *O. r. rosmarus* | Resolute Bay, NW Territories, Canada | 27 |
| USNM 35683 | NA | A | *O. r. divergens* | Port Moller, Alaska, US | 28 |
| USNM 500252 | M | SA | *O. r. divergens* | Bering Sea, Alaska, US | 29 |
| USNM 500253 | M | A | *O. r. divergens* | Bering Sea, Alaska, US | 30 |
| USNM 500254 | M | A | *O. r. divergens* | Bering Sea, Alaska, US | 31 |
| USNM 550408 | NA | J | *O. r. divergens* | NA | 32 |
| USNM 550409 | NA | J | *O. r. divergens* | NA | 33 |
| USNM 550410 | NA | SA | *O. r. divergens* | NA | 34 |
| USNM 550413 | NA | J | *O. r. divergens* | NA | 35 |
| USNM 63302 | NA | A | *O. r. divergens* | Pribilof Islands, Alaska, US | 36 |
| USNM 6780 | NA | A | *O. r. divergens* | North Pacific Ocean | 37 |
| USNM 7139 | NA | A | *O. r. divergens* | St Paul Island; Pribilof Islands, Alaska, US | 38 |
| USNM 7156 | NA | J | *O. r. rosmarus* | Greenland | 39 |
| USNM 9475 | NA | SA | *O. r. divergens* | Alaska, US | 40 |
