# Supplementary figures and images for: Sexual dimorphism in the walrus mandible: comparative description and geometric morphometrics
Source: PeerJ. 2022 Sep 20;10:e13940. doi: 10.7717/peerj.13940 (PMC9504446; doi:10.7717/peerj.13940)

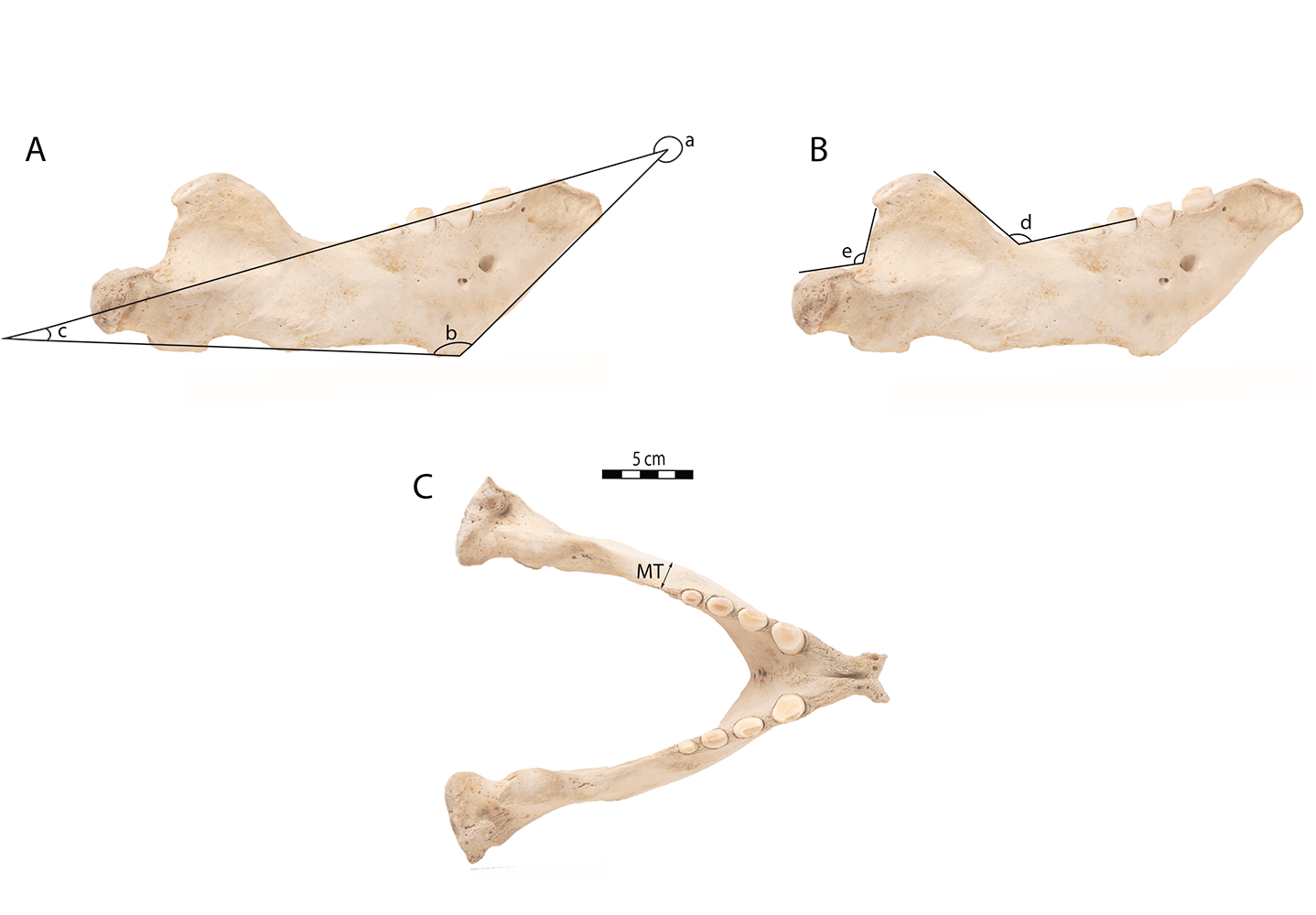

Supplement: Supplemental Information 4 — Angle between a) the anterior and dorsal margin; b) the anterior and ventral margins; c) the ventral and dorsal margins; d) the horizontal and vertical ramis; and e) the coronoid process and the mandibular condyle. MT: Least Mandible Thickness. [file peerj-10-13940-s004.png]

(1)

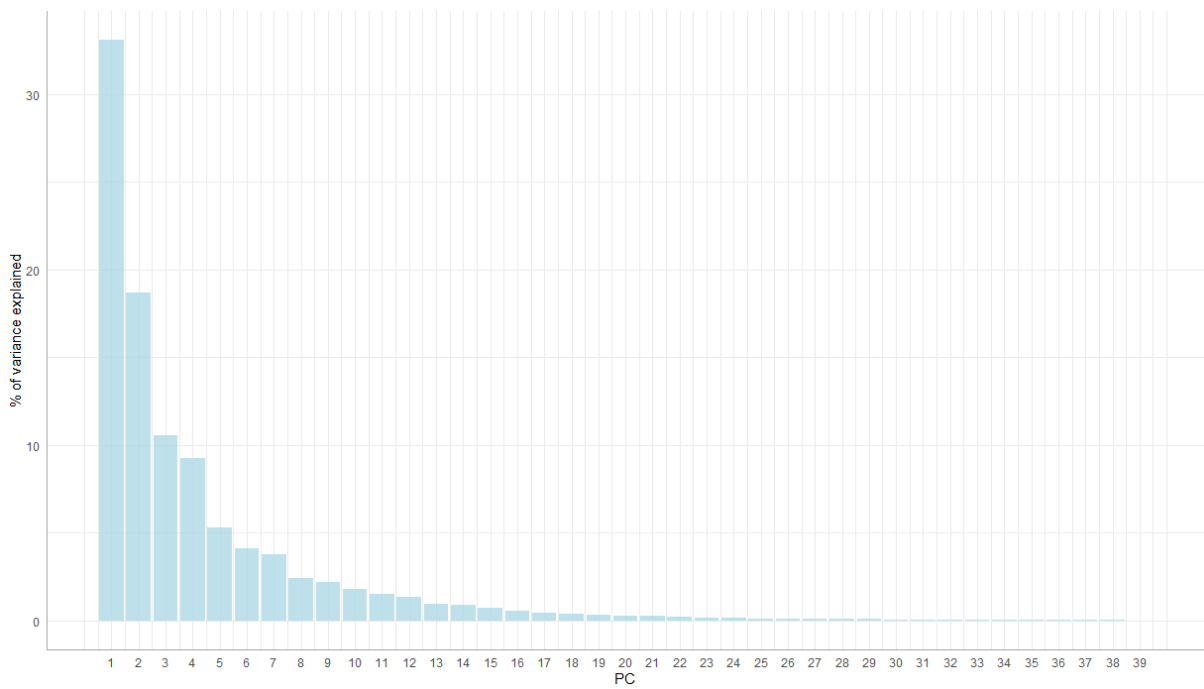

(2)

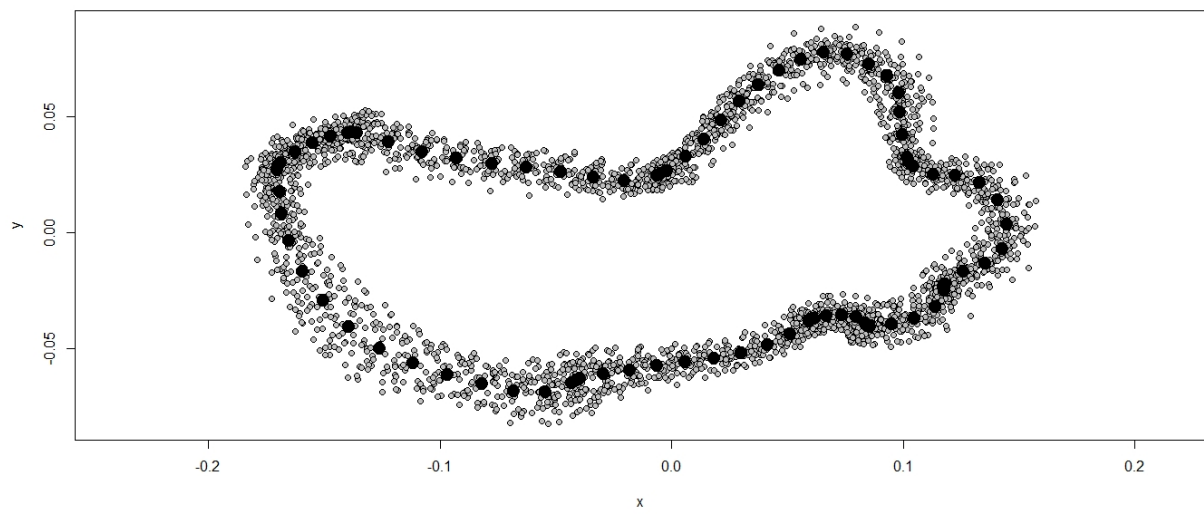

(3)

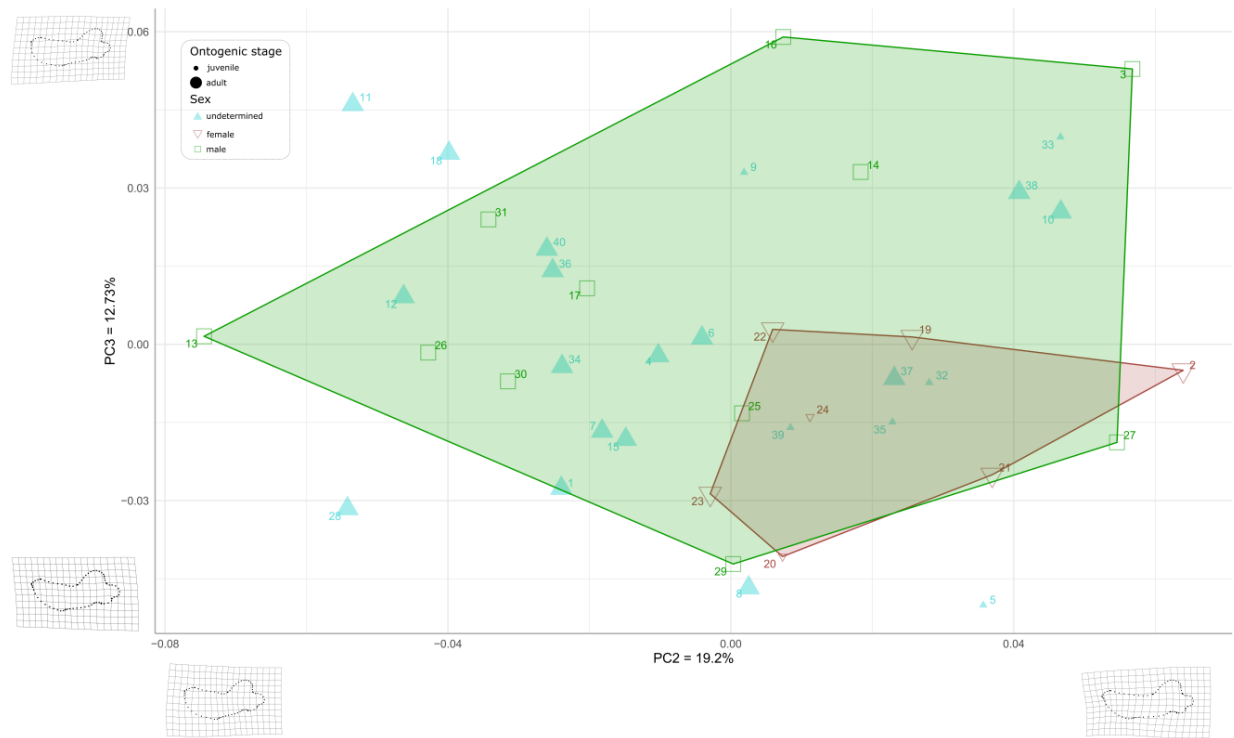

(4)

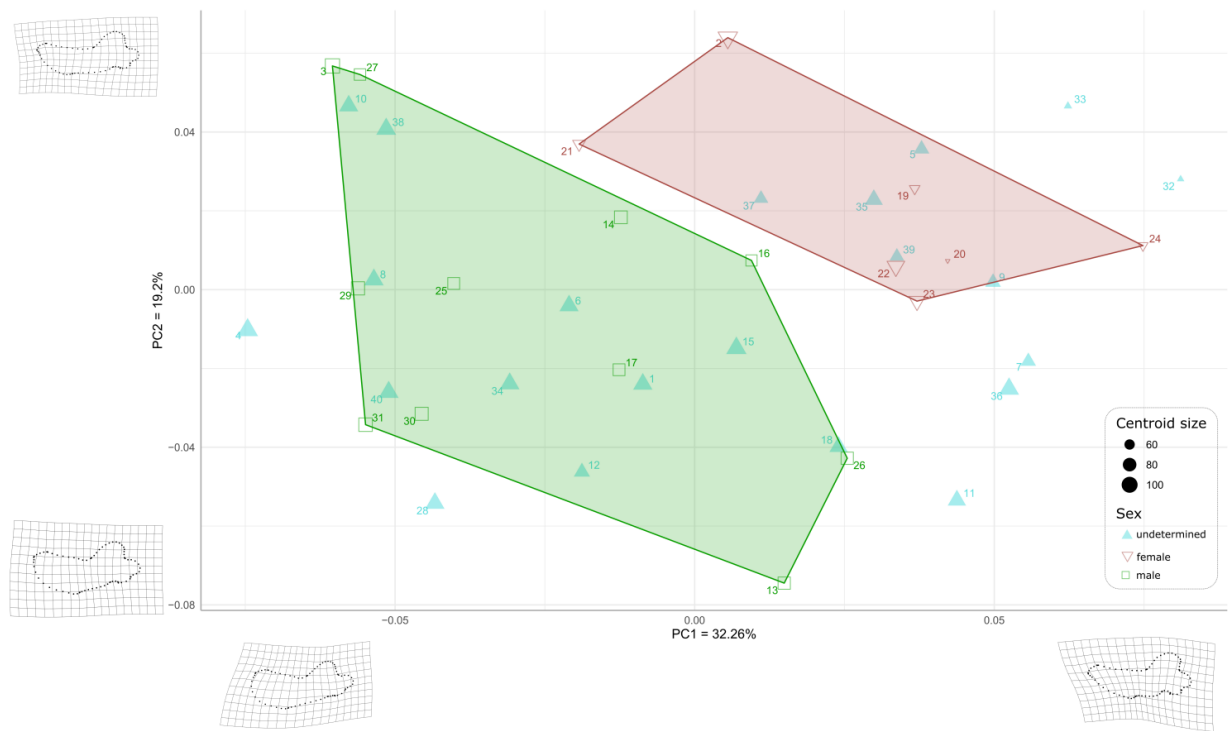

(5)

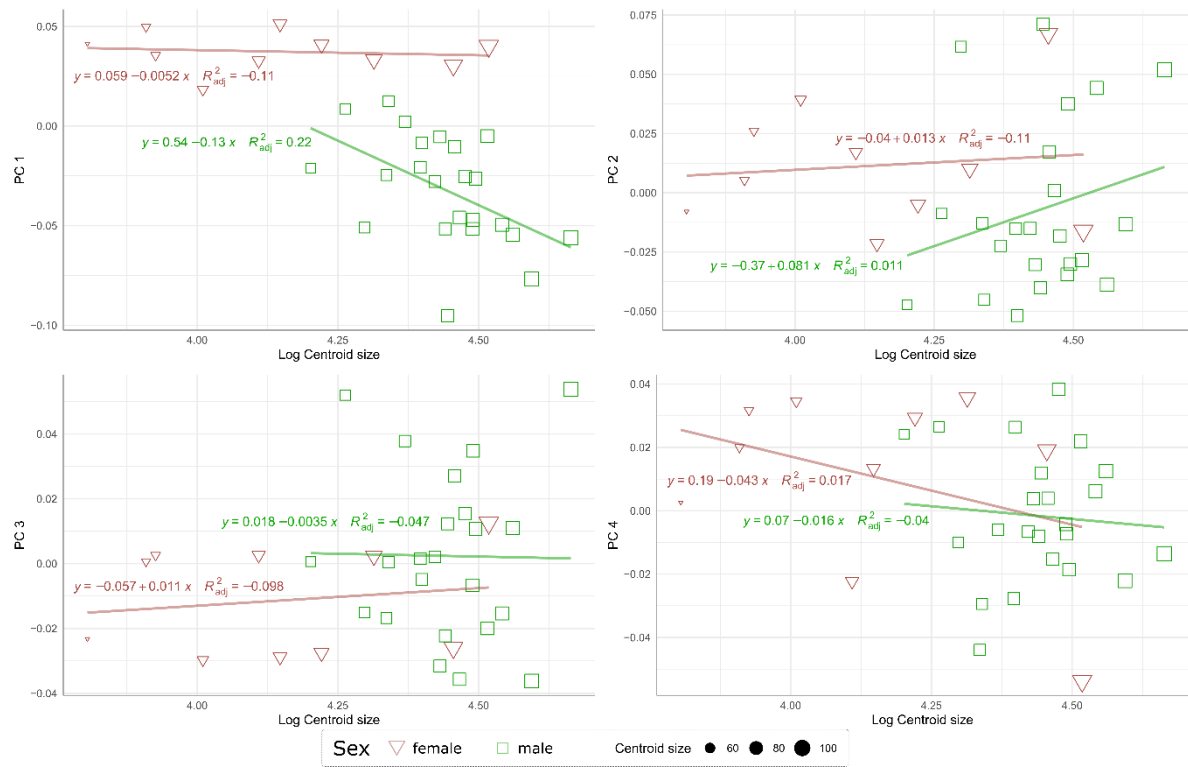

Supplement: Supplemental Information 6 — (1) Barplot showing the percentage of morphological variation explained by each of the 39 axes of the PCA. (2) Graphic representation of the general shape of the average mandible with Generalized Procrust Analysis. (3) Graph of the PCA for Odobenus rosmarus for the axes PC2 (on the x-axis) and PC3 (on the y-axis). Green square: males of O. rosmarus; red inverted triangle: females; blue triangle: sex not determined; small-size dot: juvenile; big-size dot: adult. Outlines on the upper left represent a morphotype, with features such as a shorter horizontal ramis and a significant more concave angle between horizontal vertical ramis, different from the male morphotype expressed for the lowest value of PC1 & PC2 (Figs. 7 and 8). Outlines on the lower right represent a another morphotype, with features such as a extended horizontal ramis compared to the vertical ones and a straighter ventral margin, different from the female morphotype for the highest value of PC1 & PC2 (Figs. 7 and 8). The numbers assigned to specimens follow the order etablished for the O. rosmarus specimens (Table S1). (4) Graph of the PCA for Odobenus rosmarus for the PC1 (on the x-axis) and PC2 (on the y-axis) using Log Centroid Size. Green square: males of O. rosmarus; red inverted triangle: females; blue triangle: sex not determined; small-size dot: 60; medium-size dot: 80; big-size dot: 100. Outlines on the lower left represent a male morphotype, with several features such as a straighter ventral margin, a convex anterior margin, and a more concave angle between horizontal and vertical rami. Outlines on the upper right represent a female morphotype, with several features such a slightly concave ventral margin, a straighter anterior margin, and a lower angle between horizontal and vertical rami. The numbers assigned to specimens follow the order etablished for the O. rosmarus specimens (Table S1). (5) Graph representation of regression between the log centroid size and the first fourth components o [file peerj-10-13940-s006.pdf]
