# Supplementary material for: Sexual dimorphism in the walrus mandible: comparative description and geometric morphometrics
Source: PeerJ. 2022 Sep 20;10:e13940. doi: 10.7717/peerj.13940 (PMC9504446; doi:10.7717/peerj.13940)
Supplement: Supplemental Information 5 — Letters of angles and measures correspond to those indicated in Fig. S1 [file peerj-10-13940-s005.docx]

| Characters | IRSNB 1150B (♀) | IRSNB 1150D (♂) |
| --- | --- | --- |
| Angle between anterior and dorsal margin (a) | 152 | 151 |
| Angle between anterior and ventral margin (b) | 135 | 141 |
| Angle between dorsal and ventral margin (c) | 21 | 24 |
| Angle between horizontal and vertical ramus (d) | 127 | 136 |
| Angle between the coronoid process and the mandibular condyle (e) | 116 | 98 |
| Mandibular width (MT), after the last post-canine tooth | 16.7 | 35.0 |
