# Supplementary material for: Sexual dimorphism in the walrus mandible: comparative description and geometric morphometrics
Source: PeerJ. 2022 Sep 20;10:e13940. doi: 10.7717/peerj.13940 (PMC9504446; doi:10.7717/peerj.13940)
Supplement: Supplemental Information 7 — Hypothesis H0: There is no impact of sex on the shape of the mandible [file peerj-10-13940-s007.docx]

|  | **Df** | **SS** | **MS** | **Rsq** | **F** | **Z** | **Pr(>F)** |
| --- | --- | --- | --- | --- | --- | --- | --- |
| Sex | 1 | 0.023604 | 0.023604 | 0.23766 | 4.9881 | 3.2228 | 0.001 ** |
| Residuals | 16 | 0.075711 | 0.004732 | 0.76234 |  |  |  |
| Total | 17 | 0.099315 |  |  |  |  |  |
